# Fringe Detection and Displacement Sensing for Variable Optical Feedback-Based Self-Mixing Interferometry by Using Deep Neural Networks

**DOI:** 10.3390/s22249831

**Published:** 2022-12-14

**Authors:** Asra Abid Siddiqui, Usman Zabit, Olivier D. Bernal

**Affiliations:** 1 School of Electrical Engineering and Computer Science, National University of Sciences and Technology (NUST), Islamabad 44000, Pakistan; 2 LAAS-CNRS, University of Toulouse, INP-ENSEEIHT, 31000 Toulouse, France

**Keywords:** variable optical feedback, speckle, self-mixing interferometry, laser sensing, artificial intelligence, deep learning

## Abstract

Laser feedback-based self-mixing interferometry (SMI) is a promising technique for displacement sensing. However, commercial deployment of such sensors is being held back due to reduced performance in case of variable optical feedback which invariably happens due to optical speckle encountered when sensing the motion of non-cooperative remote target surfaces. In this work, deep neural networks have been trained under variable optical feedback conditions so that interferometric fringe detection and corresponding displacement measurement can be achieved. We have also proposed a method for automatic labelling of SMI fringes under variable optical feedback to facilitate the generation of a large training dataset. Specifically, we have trained two deep neural network models, namely Yolov5 and EfficientDet, and analysed the performance of these networks on various experimental SMI signals acquired by using different laser-diode-based sensors operating under different noise and speckle conditions. The performance has been quantified in terms of fringe detection accuracy, signal to noise ratio, depth of modulation, and execution time parameters. The impact of network architecture on real-time sensing is also discussed.

## 1. Introduction

Laser-based optical feedback interferometry (OFI) or self-mixing interferometry (SMI) [1,2,3] is a promising sensing technique as it enables auto-aligned, low-cost, and contactless sensing of parameters including displacement [4], distance [5], vibration [6], velocity [7], etc. Promising results have been obtained in acoustic [8], imaging [9], fluidic [10], mechatronics [11], and biomedical [12] fields.

Reliable sensing under real-world conditions (where optical feedback strength can considerably change during the course of sensing, e.g., due to optical speckle [7] caused by non-cooperative remote target surface) is still difficult to achieve. Speckle causes measurement errors [13] and spectral broadening [14]. For displacement sensing, fringe detection (FD) is an integral part of many SMI-based measurement algorithms (e.g., using phase unwrapping [15] or non-uniform sampling [16]) and is easily achieved if the optical feedback remains constant. However, variable optical feedback (VOF) conditions make it very difficult to achieve correct FD, especially in the presence of noise. This paper presents a work that employs deep neural networks (DNNs) for FD and subsequent displacement measurement specifically under VOF conditions. By using a recently published VOF-based SMI behavioural model [17] and an automated SMI fringe labelling method, we achieve targeted training of well-established DNNs on simulated VOF-based SMI signals in order to later achieve excellent FD performance on unseen and experimental SMI signals acquired by using different SMI sensors.

**Figure 1:**
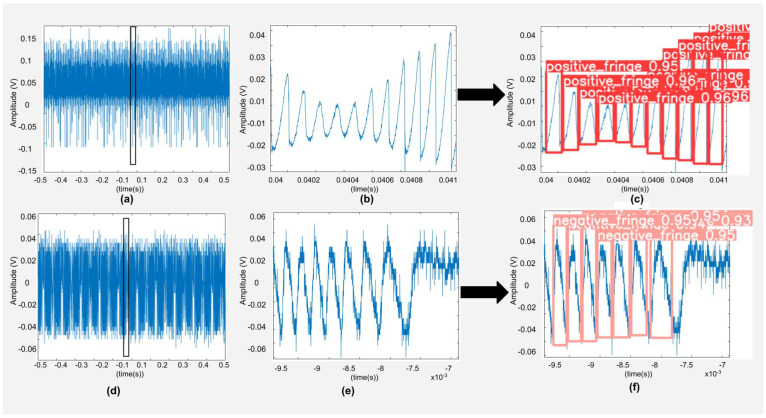
(**a**) An experimental speckle affected SMI signal from laser diode HL7851 emitting at a wavelength of 785 nm, (**b**) an enlarged view of its segment showing amplitude modulated SMI fringes, and (**c**) correct fringe detection and classification of these fringes. (**d**) A noisy experimental SMI signal emitting at a wavelength of 785 nm, (**e**) an enlarged view of its segment, and (**f**) correct fringe detection and classification using the proposed deep network.

Optical speckle [13] is encountered due to remote surface’s roughness. Interestingly, it has been used to perform sensing as well, such as for velocity [7] and flow applications [18]. However, in the context of SMI displacement sensing, speckle makes it difficult to perform FD as the SMI fringe amplitude and shape is affected (see Figure 1b) because of VOF caused by speckle [19]. Researchers have proposed different methods to mitigate the impact of speckle. For large displacements, a speckle tracking technique was reported in [20] which used a piezo-actuator to displace a focusing lens so that the laser beam avoided a dark speckle spot (causing low feedback coupling and potential loss of signal) and tracked a bright speckle spot. Further, the authors in [21] used two laser signals with different performance parameters so that SMI signal fading may be avoided. In another work, liquid lens-based control system was used to keep the optical feedback strength in a desired range [22]. However, slow response of the liquid lens could not allow real-time design and the system could only be used for initial laser spot-size setup [22]. Several digital signal processing methods have also been proposed to detect fringes under speckle’s effect such as Hilbert transform method [23], envelope extraction technique [19], and custom bi-wavelet method [24]. Similarly, an all analogue FD system was proposed in [25] to perform FD under VOF caused by speckle.

Due to immense performance improvement in neural networks during the last years, many researchers have also used these for optical sensing, such as for ultrafast machine vision with 2D material neural network image sensors [26], dual gas detection [27], optical photoplethysmography sensor [28]. Deep networks have also been used to improve computer-vision-based methods, such as for assessing steel tubular structures [29]. In the microwave domain, localization of subwavelength objects has been successfully demonstrated by artificial neural networks that appear not only robust to noise but are also capable of achieving performances beyond those obtained using the training dataset [30]. Subsequently, in the past five years, neural networks have also been used for many SMI applications, as detailed below.

One approach presented in [31] uses a single layer-based artificial neural network for classification of multi-modal SMI signals. Traditional feature extraction was used on a dataset of only 250 fringes for this network.

Another approach that uses neural networks to remove noise from SMI signal was presented in [32]. A similar work for noise removal was performed in [33] by using a generative adversarial network (GAN). In this work, the authors used a pre-processing technique to process the signals which are corrupted with white-noise and amplitude-fluctuations in all major optical regimes. They used an unlabelled, simulated training dataset obtained for different constant values of optical feedback coupling parameter, denoted as *C*, and signal to noise ratio (SNR). A one dimensional input is used. A generator is used in between an encoder and a decoder consisting of 22 convolution layers each to enhance the signal.

The work presented in [34] uses a convolutional neural network (CNN) to directly retrieve displacement from SMI signals without detection of fringes. It uses 11 layers of which 4 layers are one dimensional convolution layers, 4 max-pooling layers, and 1 dropout layer with two fully connected layers at the end. They trained their network in a restricted way because of limitation of dataset. This method appears to work well for the moderate feedback regime. Weak feedback regime SMI signals (with C< 1) were not explored. Consequently, their network cannot correctly predict displacement for small *C* cases where SMI signal tends to become symmetrical, as stated in [34].

A recent work in SMI-based vibration sensing uses joint time-frequency analysis to extract vibration information. Certain limitations in this sensing are then removed by using a generalized regression neural network (GRNN) [35].

Another work [36] uses a shallow (single hidden layer) and 1D neural network to detect the SMI fringes under constant and weak optical feedback conditions by using the fringe slope as the defining feature of a fringe. An optical attenuator was used to ensure a specific amount of optical feedback. This neural network relies on pre-processed SMI signals by using auto-correlation, amplitude-thresholding, and peak-detection operations. Detected fringes were then manually labelled (total dataset includes five SMI signals with 50 fringes each). Importantly, it does not address possible occurrence of optical speckle. Furthermore, use of fixed number of samples to represent the fringe slope restricts the method as any significant change in frequency or amplitude of remote motion needs to be measured followed by appropriate interpolation or decimation operations [36].

Another recent work by authors using a DNN for FD is currently under review [37]. This work converts 1D SMI signals to images in which SMI fringes are manually labelled for training purposes. Then, this small manually labelled dataset is vastly increased by using a novel anisotropic augmentation method that preserves the labels for augmented images. This network was trained by using constant *C*-based SMI signals, simulated by using the mathematical model reported in [38].

On the other hand, this work specifically focuses on VOF conditions. In addition to performing FD, the present work measures remote displacement along with FD by using the detected labels corresponding to correctly processed variable *C* signals. Furthermore, we remove the limitation of manual labelling of SMI fringes by proposing in this present work an automatic segmentation and labelling technique that places correct labels on the training dataset. Lastly, we have explored two different DNNs for this purpose. These networks have significantly different architectures:

- One of the models that we have implemented for FD has been recently developed by the Google Brain team, named as EfficientDet Model [39]. This model has achieved the highest accuracy with the fewest training epochs for object detection problems [40] where one epoch refers to one complete cycle of DNNs through the full training dataset.
- The other network that we have used for our variable feedback conditions is Yolov5s [41]. It is based on Darknet architecture on Pytorch framework [42]. Yolov5 is able to process 140 frames per second so can be used for real time processing of SMI [43].

We have trained both these networks by using our dataset of variable optical feedback-based SMI signals, and then tested their performance on unseen experimental SMI signals with and without optical speckle.

The rest of the paper is organized as follows. Section 2 presents a very brief overview of SMI-based displacement sensing. Section 3 presents the methodology that we have followed for this work. Performance analysis of explored networks is presented in Section 4 for simulated as well as experimental SMI signals. Section 5 presents Discussion, followed by the Conclusion.

## 2. SMI-Based Displacement Sensing

Theory of SMI is well-documented [1], and it is very briefly summarized below.

**Figure 2:**
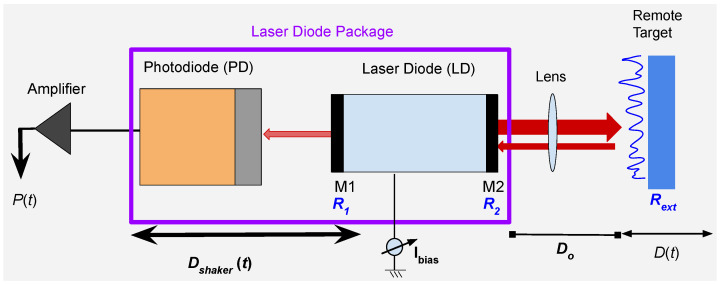
SMI laser sensor is placed at a distance $D_{o}$ from a remote target having surface reflectivity of $R_{ext}$ that is displaced at d(t). M1 and M2 are the LD mirrors having reflectivity of $R_{1}$ and $R_{2}$. The electrical signal from the monitor photo-diode is amplified and saved via a data acquisition system. SMI sensor is mounted on a mechanical shaker, vibrating at $D_{shaker}\left(t\right)$ to facilitate occurrence of speckle.

In SMI, a portion of the emitted laser beam having wavelength $\lambda_{0}$, is allowed to re-enter the active laser cavity (made up of two mirrors with reflectivity of $R_{1}$ and $R_{2}$) after back-scattering from a remote target (having surface reflectivity of $R_{ext}$) which is displaced with D(t). This causes modulation of laser power which is typically observed by a monitor photodiode (see Figure 2). The optical output power can be expressed as [1]

(1) $$P\left(t\right)=P_{0}\left(1+m\cos\left[\Phi_{F}\right]\right)\;$$

where $P_{0}$ in (1) is optical power under free-running conditions, *m* is the index of modulation, and $\Phi_{F}$ is the laser phase under optical feedback which is related to unperturbed laser phase $\Phi_{0}\left(t\right)=4\pi D\left(t\right)/\lambda_{0}$ by the relation

(2) $$\Phi_{0}\left(t\right)=\Phi_{F}+C\sin\left[\Phi_{F}+arctan\left(\alpha \right)\right]\;$$

where *C* is the optical feedback coupling factor that defines the operating regime, and α is the laser line width enhancement factor.

In the absence of fringe-loss, each interferometric fringe in the SMI signal is assumed to occur after every $\lambda_{0}$/2 target displacement. Thus, a simple fringe counting algorithm provides a resolution in displacement measurement of $\lambda_{0}$/2 [38]. Advanced phase unwrapping algorithms [44] require FD and provide better performance by solving (2) after estimating *C* and α parameters [45].

### Variable Optical Feedback Conditions

The *C* parameter depends upon the amount of feedback, surface reflectivity, coupling efficiency, and the distance to the target [16]. The shape of OFI signals and value of modulation index *m* depend on *C* [25]. The modulation index can be expressed by [1]:

(3) $$m=\frac{2\tau_{p}}{\tau_{c}}\epsilon \sqrt{R_{ext}}\frac{1-R_{2}}{\sqrt{R_{2}}}\;=\;C\frac{\tau_{p}c}{L\sqrt{1+\alpha^{2}}}$$

where *C* is given by:

(4) $$C=\epsilon \sqrt{R_{ext}}\frac{1-R_{2}}{\sqrt{R_{2}}}\frac{L\sqrt{1+\alpha^{2}}}{l_{cav}n}$$

Here, ϵ is the coefficient that caters for possible mode mismatch between the lasing mode and the reflected light, $\tau_{p}$ represents the lifetime of photons within the cavity, $\tau_{c}$ is the round trip time taken by light within the interval cavity, $l_{cav}$ is the length of laser cavity, *n* is the refractive index of cavity [46], $R_{ext}$ represents the reflectivity of external target, and $R_{2}$ is the reflectivity of laser front mirror that emits the light. *L* is the distance between the laser and the remote target [1].

Depending on the value of *C*, specific SMI operating regimes have been identified [1], summarized below:

- C<0.1: Very weak feedback regime where P(t) has an almost sinusoidal shape. Consequently it is very difficult to distinguish fringes corresponding to forward motion from fringes corresponding to backward motion.
- 0.1<C<1: Weak feedback regime where P(t) has an asymmetrical and increasingly sawtooth-like shape.
- 1<C<4.6: Moderate feedback regime where P(t) has hysteresis in it and the fringe shape is sawtooth-like. This regime is widely used for metric sensing due to ease of FD and a direct identification of motion direction by the shape of the fringes.
- C>4.6: Strong feedback regime with appearance of fringe-loss.

## 3. Methodology

In this work, we explore object detecting DNNs to detect and classify SMI fringes. These networks first perform FD and then classify each detected fringe in two classes: positive fringe (corresponding to forward motion) and negative fringe (corresponding to backward motion). To train our networks for fringe detection of variable optical feedback-based SMI signals, first we have to create a dataset that includes many SMI signals under different variable optical feedback conditions.

**Figure 3:**
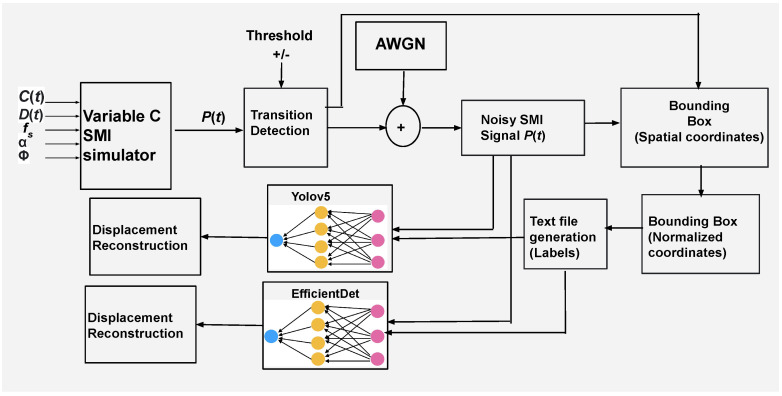
Schematic block diagram of complete system including generation of VOF-based SMI signals, proposed fringe labelling method, training of neural networks and displacement reconstruction. After training, unseen experimental signals were used for testing. AWGN denotes additive white Gaussian noise.

The schematic block diagram of the complete system is shown in Figure 3. First, we generate noiseless SMI signals using a variable *C*-based SMI simulator [17]. Noise is then added to these signals. We have used the additive white Gaussian noise (AWGN) model to represent the typical experimental noise affecting the SMI signals [47]. A typical SMI signal that we used comprises 100 k samples. This typical SMI signal is converted into 140 images. In the next step, we perform automated bounding box placement and labelling of fringes contained inside these SMI signals’ images. These labels and SMI signals’ images are fed to DNNs to train them. Our proposed work uses DNNs including Yolov5s and EfficientDet (detailed ahead) to correctly detect and classify multiple fringes present in an image at a frame-rate of 140 frames per second. At the end, displacement is reconstructed based on output of these models.

The next subsections will explain these steps of dataset generation for variable optical feedback conditions, automated labelling, training of our DNN models, and displacement reconstruction.

### 3.1. VOF-Based SMI Signal Generation and Automated Labelling

In this subsection, generation and automatic labelling of variable optical feedback SM signal is explained.

**Figure 4:**
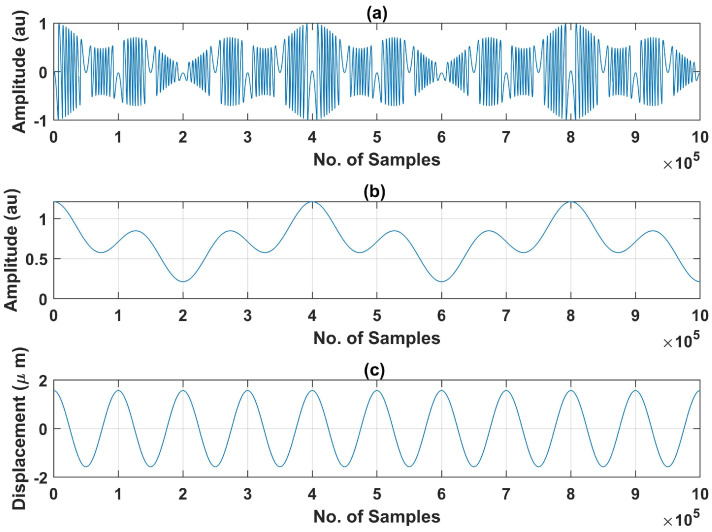
Example of generated SMI signals used in the training dataset: (**a**) generated SMI signal of one second duration with $f_{s}$ of 100 k samples/s corresponding to variable *C* shown in (**b**) and remote target motion D(t) shown in (**c**).

The training dataset that we have created includes SMI signals having different continuously varying spans of *C* (one such signal is shown in Figure 4). For training data, *C* values range from 0.5 to 8 and SNR was varied from 15 dB to 22 dB. For all these SMI signals, we created a label file (containing the class and bounding box of each fringe existing in the corresponding image of the SMI signal) in an automated manner.

**Figure 5:**
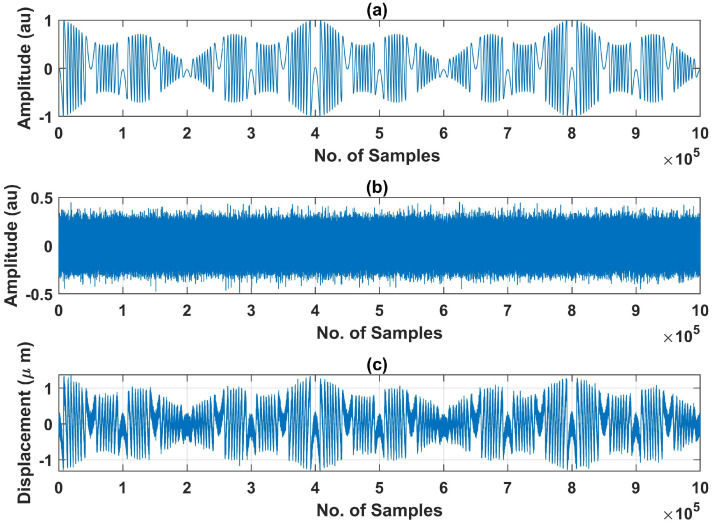
Addition of noise to noiseless variable *C*-based SMI signal: (**a**) noiseless SMI signal (**b**) noise signal, and (**c**) noisy SMI signal obtained by addition of the noiseless SMI signal and the noise signal.

One such signal with additive noise is shown in Figure 5.

We have generated these SMI signals in MATLAB by using the variable *C*-based SMI simulator [17]. It requires information about remote motion’s frequency, peak to peak amplitude, initial phase ϕ, sampling frequency $f_{s}$, and variation in *C*. We have used multiple vibration frequencies for training dataset ranging from 2 Hz to 10 Hz and $f_{s}$ was set to 100 kHz. Furthermore, different peak to peak amplitude values, ranging from 2 to 8 μm, were used. For C(t), we used different variations including sinusoidal and arbitrary variations.

Next step is to detect transitions of this SMI signal. For this purpose, we use the simple derivative-based method proposed in [48] to detect the transitions (indicative of fringes) in the simulated and noiseless SMI signal. In this method [48], derivative is compared with a positive and a negative threshold to detect a positive or a negative fringe. This simple derivative-based FD method is giving correct FD results for simulated VOF conditions only in absence of any noise. Any significant presence of noise results in poor FD performance by this method as it was proposed for constant and moderate feedback regime only [48]. Next step is to add noise in this SMI signal, as shown in Figure 5.

After the noise is added, the corresponding SMI signal is processed to place bounding boxes on its fringes in an automated manner by using the transition value (1 and −1) as class identity (ID).

#### Bounding Box Placement on SMI Fringes

**Figure 6:**
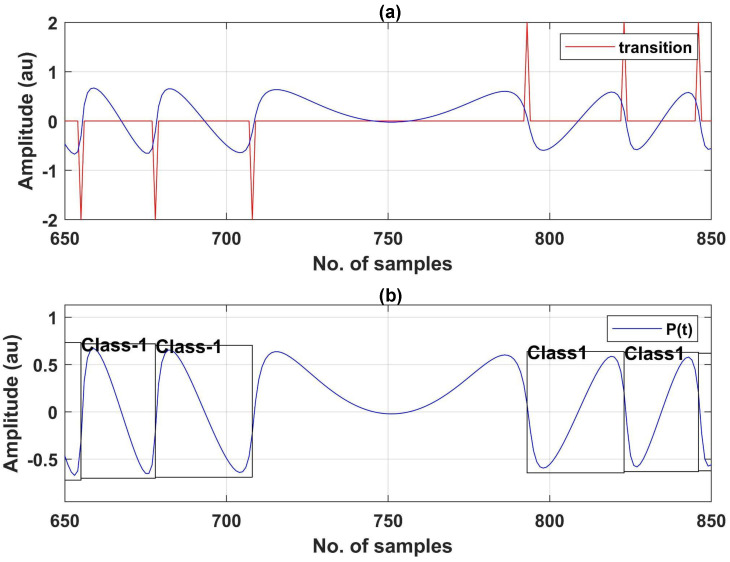
Automated labeling of SMI signals: (**a**) transitions detected on a simulated and noiseless SMI signal. (**b**) Automated placement of bounding box and class label on the SMI fringes.

This subsection describes how automated placement of bounding boxes on each SMI fringe is achieved. The steps required are summarized below as:

1 Find x coordinates of bounding box of each fringe by using information provided by the transition values (see Figure 6a).
2 Find y coordinates of bounding box of each fringe by using local minimum and local maximum value search, performed in the x coordinates range found in first step.
3 Avoid the bounding box placement on hump regions (where motion direction reversal occurs) by using the information of transition values.
4 To make the bounding boxes in accordance with the input format requirement of DNNs, required parameters are found and normalization of coordinates is carried out.

Now we shall explain in detail all the steps stated above.

First step in making a bounding box is to find x coordinates for the box. To find x coordinates, i.e., starting point of fringe $x_{1}$ and end point of fringe $x_{2}$ for bounding box of fringe, we use transition values as markers. In order to locate the position of fringe, we find the indices of transition and respective class of transition and save these in two different dictionaries.

Next, in order to find y coordinates of the bounding box, we search for local minimum and local maximum of SMI signal within $x_{1}$ and $x_{2}$. This provides $y_{min}$ and $y_{max}$ for a single fringe. Consequently, ($x_{1}$,$y_{min}$) corresponds to the left-bottom of the box, and ($x_{2}$,$y_{max}$) corresponds to the top-right of the bounding box.

To avoid placing a bounding box on any hump region, we identify the regions of hump by searching for any change in sign of transition value between any two consecutive transition values. This identifies the humps because when there is a hump, direction reversal of SMI fringe occurs.

Now, we place a bounding box with information of $x_{1}$, $y_{min}$, width, and height where width corresponds to difference of $x_{2}$ and $x_{1}$ and height corresponds to difference of $y_{max}$ and $y_{min}$. Note that these spatial coordinates are of the 1D SMI signal. For object detectors, we need to move from 1D spatial coordinates to normalized coordinates. These object detector labels require the information of bounding box in a text file in format of columns where each column represents class, $x_{center}$$y_{center}$, width and height, respectively. To transform our spatial coordinates, we normalize x-axis and width by total number of samples while we normalize y-axis and height by amplitude. $x_{center}$ is found by $x_{1}$ + width/2 and $y_{center}$ by $y_{min}$ + height/2. At this point, we have the information that is required to be saved in a text file, as needed by object detectors. The labels on each fringe appear as shown in Figure 6.

The label file contains information of bounding boxes and class name in column format. Yolov5 model uses .txt annotation for the label file containing the information of bounding boxes. EfficientDet model uses these text files to generate a large annotation file of complete dataset. So we needed to devise a method that can generate an SMI signal along with the corresponding .txt file. Once all the dataset is generated and labelled, we use augmentation techniques to expand our dataset. We have used vertical and horizontal concatenation for our signals and their corresponding labels. Augmentation of datasets is performed because these machine learning models perform very well for larger datasets and result in better accuracy. Now, our dataset is complete for training on Yolov5.

To train our data on EfficientDet model, we reuse the same dataset. EfficientDet model uses another label format called .json for training. So, to use this dataset and its labels, we have to convert .txt format to .json format so that we can use the same dataset and label files for our EfficientDet model. An annotation file in .json format was created for our complete augmented dataset. We have partitioned our dataset in training and validation folders. Annotation files for both training and validation datasets are created. For EfficientDet model, we shall use training images, their respective annotation file, validation images, and their annotation file.

### 3.2. Fringe Detection

After our dataset is complete, the next step is to train DNN models in order to detect fringes in a noisy VOF-based SMI signal. As previously stated, we have implemented two models namely EfficientDet D0 and Yolov5s for this purpose. These two models have different architectures, summarized below.

#### 3.2.1. EfficientDet Model

**Figure 7:**
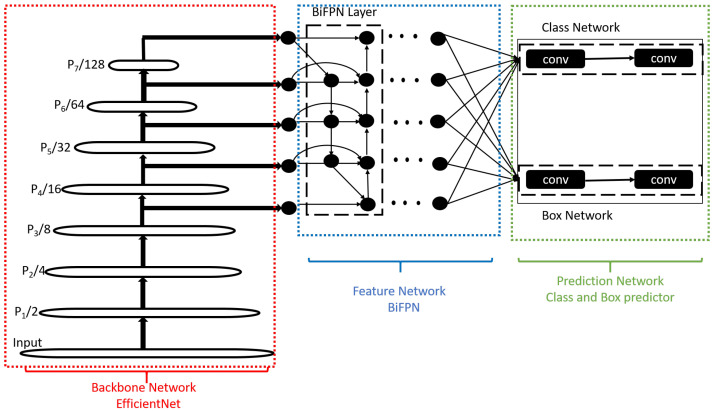
The architecture of EfficientDet model [39].

EfficientDet model [39] employs ImageNet pre-trained EfficientNet as backbone network having 5 times faster performance on CPU and 2 to 4 times faster on GPU as compared to other models. EfficientDet uses compound scaling and Bi-directional Feature Pyramid Network (BiFPN) that takes features from backbone levels 3 to 7 and then repeatedly applies bidirectional feature fusion in both directions, i.e., top-down and bottom-up approaches. The resulting fused features serve as an input to the next network that produces class and box prediction of the object. It uses multiple convolution networks (conv) for prediction. The block diagram of EfficientDet Network is shown in Figure 7. EfficientDet Model is reported to make better use of resources and provides better accuracy [43].

#### 3.2.2. YOLOv5

**Figure 8:**
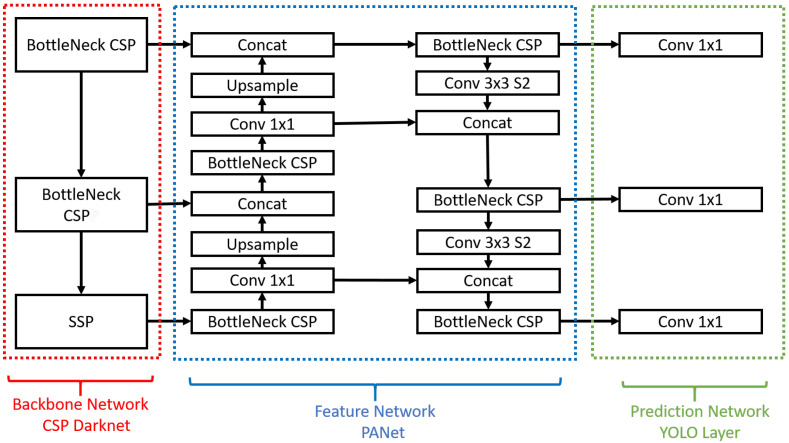
The architecture of Yolov5 model [41].

Yolov5 developed by Ultralytics [41] uses Bottleneck Cross Stage Partial (CSP) Networks as backbone to extract features from an input image by repeated convolution and pooling layers. Yolov5 uses Spatial Pyramid Pooling (SSP). This SSP and bottleneck CSP help improve accuracy of detection by extracting features from different scales. It also uses a class and box prediction network at the end called as Yolo Layer to classify and localize the object. This network uses multiple Conv Nets for this purpose. The network architecture of Yolov5 is shown in Figure 8.

The research community continues to explore and improve these architectures. For example, a recent work [49] uses Yolov4tiny object detector model. In it, a variant named as Yolo-Oleifera has been proposed to deal with the challenges of mobile-robot-based detection and location of fruit called as Camellia Oleifera. This modified version of Yolov4tiny has shown better results for fruit detection in a complex real orchard environment under different sunlight and shading conditions.

#### 3.2.3. Fringe Detection for Both Models

**Figure 9:**
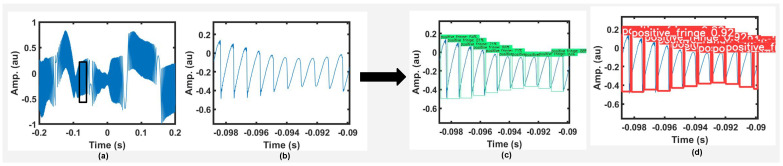
Fringe labeling applied on a speckle affected experimental SMI signal (**a**), (**b**) an enlarged view of a segment of this SMI signal, (**c**) FD results by the trained EfficientDet model, and (**d**) by the trained Yolov5 model.

We have used EfficientDet D0 model to train and detect fringes for variable feedback-based SMI signals. We have trained model on simulated data with a batch-size of 8. The batch-size indicates the number of samples that are processed in one pass, be it forward or backward during training of the model. There were 130,000 images in our complete augmented dataset. We have tested our model on experimental data that is unseen for the model shown in Figure 9. For training, we have used simulated signals while for testing, we used unseen experimental signals.

We have implemented and trained a Yolov5s model on our dataset of Variable feedback conditions. Yolov5 model takes images and its corresponding labels in .txt format as training data. We trained our model by using a batch-size of 8. The time taken by this model was 2 iterations per sec. For one epoch, this model took 2 h to train.

Similarly to the EfficientDet D0 model, this trained Yolov5s model is then tested on experimental data that was not used in training at any stage. Our trained model performed very well on these signals and correctly predicted and classified fringes as positive and negative fringe. This prediction on one of the speckle affected, amplitude modulated SMI signal is shown in Figure 9.

Once the model is trained, we tested VOF-based speckle affected experimental signals to check the performance. We noted that both the models can process 140 frames per second in terms of fringe detection and classification. After fringes are detected, displacement reconstruction is performed by using generated labels of fringes, as detailed below.

### 3.3. Displacement Reconstruction

The next step is displacement reconstruction by using the detected positive and negative fringes. We have used fringe-counting method having resolution of λ/2 to show that we can retrieve displacement information from the output of the DNNs. Better measurement precision can be obtained by using one of advanced algorithms, such as by direct phase unwrapping [48] or local phase inversion correction [45] or non-uniform sampling-based motion retrieval [50] all of which need correct FD to proceed. It could even be worthwhile to use a neural network to extract displacement with better precision by using the FD information that our DNNs provide, in a manner similar to [34].

**Figure 10:**
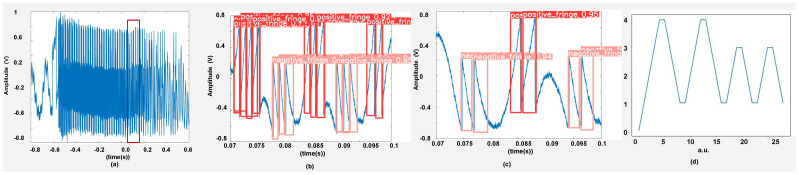
Displacement reconstruction for 2 segments of an experimental SMI signal: (**a**) experimental signal, (**b**,**c**) show two consecutive segments of the signal located in the red rectangular box of (**a**), and (**d**) shows the reconstructed displacement based on labels provided by the DNN.

To extract displacement, we save the labels generated by the neural network after it has processed an SMI signal. We then compile these label files in a .csv file. This .csv file has labels saved in the form of 1 and −1 for two classes of positive and negative fringe, respectively. These values are then read in a separate file and then passed through an integrator to obtain displacement, with a resolution of λ/2. This completes the task of measuring remote target’s displacement even under the influence of optical speckle. An example is shown in Figure 10.

## 4. Results

We have trained our dataset on Yolov5 and EfficientDet models for weak and moderate feedback regime such that values of *C* vary from 0.5 to 8 and value of SNR varies from 15 dB to 22 dB with a sampling frequency of 100 ksamples/s. Both the models use same dataset with same characteristics of SMI signals for training. We have also used same image size for both the models so that we can fairly compare their performances. The next subsections will explain simulated and experimental results on unseen data.

### 4.1. Simulated Results

**Figure 11:**
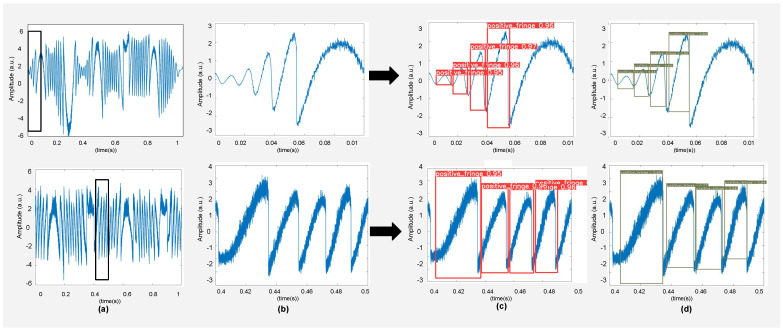
Results of testing of simulated SMI signals are shown on trained DNN models. (**a**) shows variable *C*-based simulated SMI signals under similar conditions of training, (**b**) shows an enlarged view of a segment of this SMI signal, and (**c**,**d**) show the results obtained by YOLOv5, and EfficientDet, respectively.

We have measured the performance on simulated data to quantify the range of *C* and SNR for correct detection and classification of fringes, as shown in Figure 11. We have obtained these SMI signals under simulated environment similar to the one that we have used to generate our training data. The trained models correctly detected and classified fringes contained in simulated noisy and variable *C*-based signals. The results for these simulated data are shown in Table 1.

**Figure 12:**
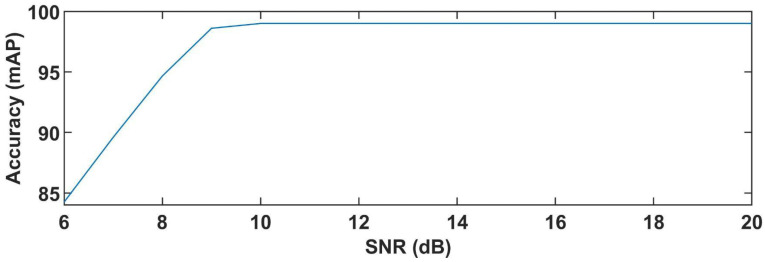
Fringe detection accuracy provided by the DNNs as a function of SNR of simulated noisy SMI signals. *C* for these SMI signals varies in the range from 0.4 to 6.

We have also quantified the relationship between SNR of noisy SMI signals with variable *C* and fringe detection accuracy of these methods. We have tested various simulated SMI signals under variable optical feedback conditions subjected to different levels of noise. We have used the SMI signals with SNR values from 6 dB to 20 dB while variation in *C* ranges from 0.4 to 6. As we can see from the plot shown in Figure 12, when the SMI signal is very noisy, i.e., SNR is lower than 9 dB, the fringe detection accuracy measure tends to drop. As the plot shows that, the accuracy drops to 84.25% for SNR of 6 dB. When the SMI signal has relatively better SNR (9 dB and above) the fringe detection accuracy tends to approach 99%.

### 4.2. Experimental Results

**Figure 13:**
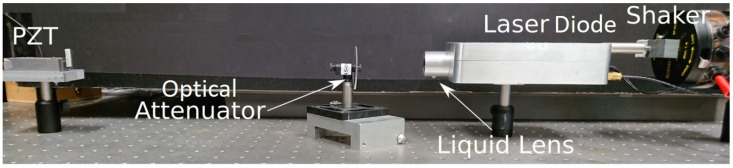
Photograph of the experimental setup for SMI-based sensing.

**Figure 14:**
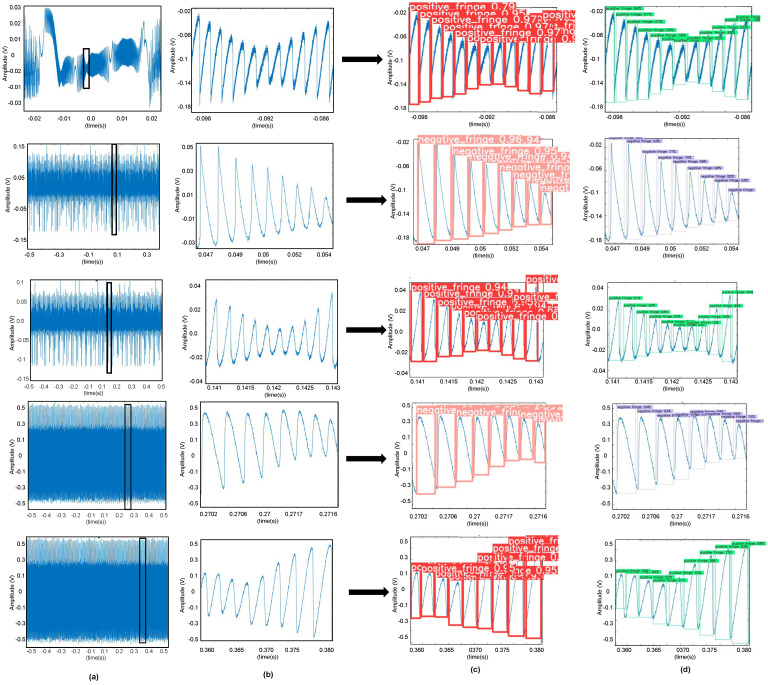
Results of testing of trained DNNs on speckle affected experimental SMI signals: (**a**) SMI signals, (**b**) enlarged view of a segment of the SMI signal, and (**c**,**d**) show the results provided by YOLOv5 and EfficientDet, respectively.

**Figure 15:**
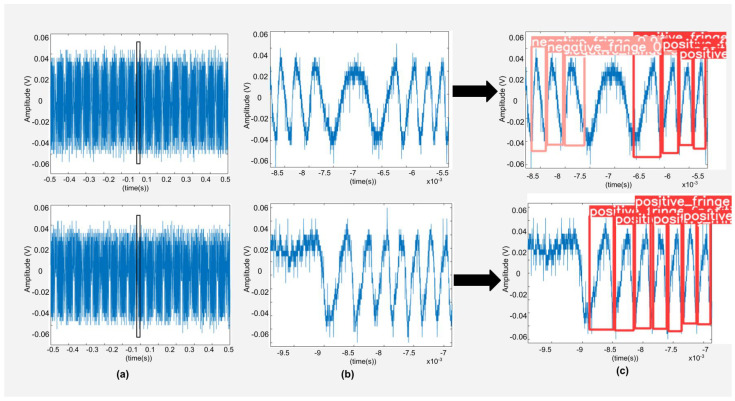
Results of testing of noisy experimental SMI signals with low SNR (**a**) shows the SMI signals (acquired by using experimental setup number 3) with commercial laser diodes emitting at 785 nm with bias current of 76 mA and 36 mA while liquid lens voltage was set to 45 mV for both signals, respectively. (**b**) is the enlarged view of a segment of SMI signal (**c**) shows the correct FD for corresponding laser diode SMI signals.

To show the robustness and generalization of this work, we have used different experimentally acquired SMI signals. These experimental signals were acquired from experimental setup schematized in Figure 2 and photographed in Figure 13. A piezoelectric transducer (PZT) device was used as remote target. We have used three different settings from the same setup to acquire signals. These different configurations are summarized as:

1 We have used the setup with the conditions of vibrating target where $D_{shaker}$ is 0 and standard lens is used. $I_{bias}$ in the setup is constant for this case. One signal, acquired under these conditions, is shown in Figure 9.
2 To facilitate occurrence of speckle, shaker, and target both are vibrating and standard lens is used. $I_{bias}$ in the setup is kept constant for this case. The signals obtained from such a configuration are shown in Figure 14.
3 We added a liquid lens [24] in front of the standard lens, shown in Figure 13. We also varied $I_{bias}$ in a step-wise manner. $D_{shaker}$ is set to 0. Variations in $I_{bias}$ and liquid lens voltage resulted in obtaining SMI signals with different SNR characteristics, shown in Figure 15.

The remote motion’s characteristics in terms of vibration frequency and peak-to-peak amplitude of the PZT device are mentioned in Table 2.

In Figure 14, we present different experimental speckle affected SMI signals. We have shown an enlarged view of one segment of each of these SMI signals in Figure 14b. Each segment shows variable optical feedback conditions where *C* value and signal amplitude is varying. As can be seen from Figure 14c,d, both the trained DNNs are able to correctly detect and classify positive and negative class fringes under experimental VOF conditions.

We have also tested the SNR range for which we are able to obtain the correct detection of fringes. We have used signals from two different commercial laser diodes for this purpose (see Figure 15) whose specifications and lower SNR limit for correct detection are shown in Table 3. The DNNs are able to correctly detect signals with SNR as low as around 9 dB despite significant noise present in the signals. This can be seen in Figure 15c where correct detection is achieved for signals having SNR of 9.19 dB and 10.06 dB, respectively. Below such SNR values, incorrect detections occur. So, we can define the lower limit of correct detection up to SNR value of 9 dB for the present work.

**Figure 16:**
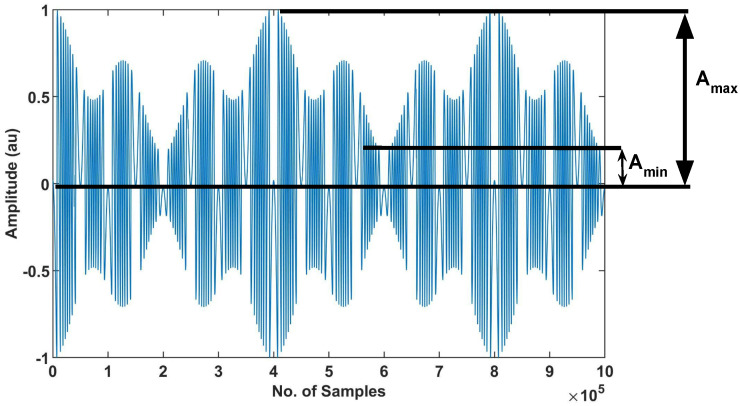
$A_{max}$ and $A_{min}$ are shown for a simulated VOF-based SMI signal.

To further quantify the impact of speckle on SMI signals, we estimate the depth of modulation parameter, denoted as $\hat{m}$, for our experimental speckle affected SMI signals. It indicates how much the amplitude of SMI fringes has varied within the same SMI signal, caused by optical speckle. It is defined in Equation (5) by using $A_{max}$ and $A_{min}$, where $A_{max}$ denotes the amplitude of the tallest fringe while $A_{min}$ denotes the amplitude of the smallest fringe within the SMI signal. For an SMI signal, $A_{max}$ and $A_{min}$ are shown in Figure 16.

(5) $$\hat{m}=\left(A_{max}-A_{min}\right)/\left(A_{max}+A_{min}\right)$$

We have reported in Table 2 the maximum depth of modulation for our experimental SMI signals affected by speckle.

### 4.3. Performance Analysis

As we trained both models with the same dataset, we can compare the training time that both these models consumed for our dataset. Our dataset was divided into training and validation datasets. Total images after augmentation were 130,000. We have trained both of these models on NVIDIA GPU GE FORCE RTX 2080 8GB by using the same batch-size of 8. The impact of network architecture of both the detectors can be explained in terms of training time and accuracy. Batch-size and training time have inverse relation. Higher batch-size leads to smaller time but very high batch-size may negatively affect the generalization of the model. So, we used the batch-size of 8 for our training. Hence to complete one epoch on dataset of 130,000 images with a batch-size of 8 means that there would be 16,214 iterations (130,000/8) in one epoch. We trained both the DNNs for 60 epochs. For Yolov5, the training time that it took was 1 iterations per second and the time consumed was 4 h approximately for one epoch. Hence Yolov5 took total time of 240 h for 60 epochs. For EfficientDet model, the training time was 2 iterations per second for same batch-size. So, the total time for one epoch was approximately 2 h. It took around 120 h to complete training for 60 epochs. Hence, we can conclude that EfficientDet requires less training time as compared to Yolov5 for our augmented dataset. This difference in training time is due to compound scaling and BiFPN that EfficientDet uses [43].

We have also measured the inference time for both DNNs. The inference time is the time taken by the trained model to process (i.e., infer the result of) a test image. On average, Yolov5 took 0.249 s per image, for inference. EfficientDet took around 0.03 s per image, for inference. Hence, we can conclude that EfficientDet takes less time for training and inference, as can be seen in Table 4.

Another performance measuring metric that is widely used is mean Average Precision (mAP). mAP measures the score from comparison of ground truth bounding box (true label) with the predicted bounding box. Higher value of mAP refers to higher accuracy of the model under observation. The mAP value measured for Yolov5 was 0.991. For positive fringe class the mAP value was recorded as 0.995 while for negative fringe class it was about 0.987. For EfficientDet the mAP value measured is about 0.943. For positive and negative class it was measured to be around 0.948 and 0.936, respectively. We noted a small difference in accuracy of Yolov5 and EfficientDet models that is mainly because of the architectural difference that comes with a trade off between accuracy and time consumption of the two models as shown in Table 4. Better accuracy of Yolov5 is mainly due to the use of SSP and bottleneck CSP (see Section 3.2.2 for Yolov5’s architecture details) that helps to improve accuracy of detection by extracting features from different scales.

## 5. Discussion

In this paper, we have proposed a method for automatic labelling of our dataset of SMI signals. We have trained and tested two models namely EfficientDet and Yolov5, for fringe detection in case of variable optical feedback conditions. We have also retrieved displacement for the case of Yolov5 by using the labels that it creates for test signals for fringe detection and classification of variable *C*-based SMI signals. Both the models performed well on the unseen test data. EfficientDet model has shown significant training time reduction by approximately 50 percent as compared to Yolov5.

It is recalled that SMI is based on a laser under optical feedback. This results in a nonlinear relation between the remote displacement and the laser power signal, relation that is strongly affected by speckle that induces changes in optical feedback and thus in OFI fringe shape. As a result, the aim of our work is not to propose the best neural network to address the fringe detection issues encountered in optical feedback interferometry that often limit the OFI sensor robustness performances. Instead, we show that using a neural network such as Yolov5 is a good choice to efficiently tackle this issue, thereby allowing a more broad usage of OFI sensors. A thorough discussion of different neural networks to deal with this fringe detection issue is beyond the scope of our present work.

Another work that is under review [37] also uses Yolov5 model to detect fringes. The major difference is that we propose a method for automated labelling of fringes contained in SMI signals and then after performing FD, we measure displacement thereby providing an end to end solution for displacement measurement from SMI signals. Furthermore, the work in [37] uses a dataset containing non-variable optical feedback regime SMI signals in which the *C* value remains constant whereas we have dealt with VOF-based signals in which the amplitude and shape of SMI fringes varies over time, even within a single SMI signal. Although this model [37] also shows fair accuracy for moderate and weak feedback SMI signals but this occurs only when the feedback regime does not change over time during SMI signal acquisition. This work [37] shows cases of incorrect detection and classification when the SMI signal is significantly affected by speckle. Another aspect that is different is in terms of segmentation of SMI signals. We have used automated labelling instead of manual labelling used in [37]. We have performed the segmentation of SM signals automatically whereas this work [37] uses manual segmentation in which each SMI signal is treated differently. Each segment is made by using the mean amplitude of that segment as there is a deep variation in amplitudes of speckle affected signals. Our work uses automated segmentation of speckle affected SMI signals. We have tested this model [37] on our test data where it shows significant degradation in accuracy in case of speckle affected experimental SMI signals (see Table 5).

## 6. Conclusions

In this paper, we have trained and tested two deep neural networks for fringe detection under noisy variable optical feedback conditions. We have also proposed an automated fringe labelling method for such SMI signals so that time-consuming and tedious operation of manual labelling is no more needed to train these deep neural networks. We have also retrieved displacement from the results of fringe detection by using the output labels provided by the networks. For unseen experimental SMI signals, these networks performed correct fringe detection for SNR as low as 9 dB and for maximum depth of modulation of 96%, caused by speckle. We have discussed the performance of both the networks in terms of training-time, inference-time, and mAP (accuracy) parameters. We have found that Yolov5 network provides better accuracy of 99.1% as compared to 94.3% for EfficientDet. On the other hand, EfficientDet has a smaller training time (2 iterations per second) and inference time (0.03 s) compared to Yolov5’s training-time (1 iteration per second) and inference time (0.249 s).

## Figures and Tables

**Table 1:** Performance of both DNNs in terms of detection and classification as a function of *C* and SNR for simulated data.

*C* Variations (Training Dataset)	Noise Variations (Training Dataset)	*C* Variations (Simulated Test Data)	Noise Variations (Simulated Test Data)	Correct Fringe Detection (Simulated Test Data)	Correct Fringe Classification (Simulated Test Data)
0.5 to 8	15 dB to 22 dB	1.5 to 6	22 dB	Yes	Yes
-	-	0.8 to 5	26 dB	Yes	Yes
-	-	0.5 to 4	21 dB	Yes	Yes
-	-	0.4 to 3	22 dB	Yes	Yes
-	-	1.5 to 6	14 dB	Yes	Yes
-	-	0.8 to 5	10 dB	Yes	Yes
-	-	0.5 to 4	12 dB	Yes	Yes
-	-	0.4 to 1.5	13 dB	Yes	Yes

**Table 2:** Specifications of different parameters under which experimental SMI signals were acquired including the estimated depth of modulation, $\hat{m}$, caused by optical speckle.

Wavelength	PZT Frequency	PZT Vibration Amplitude	Shaker Frequency	Depth of Modulation Caused by Speckle for Correct Detection
785 nm	80 Hz	10 μm	90 to 120 Hz	95%
785 nm	40 to 60 Hz	25 μm	100 to 110 Hz	96.6%

**Table 3:** Typical values of commercial laser diodes used for performance analysis of signals obtained as function of SNR.

Laser Diode	Wavelength	Typical Threshold Current	Min SNR Value for Correct Detection
DL7140	785 nm	25 mA to 60 mA	10.40 dB
HL7851	785 nm	45 mA to 70 mA	9.19 dB

**Table 4:** Comparison of inference time, training time, and mean Average Precision (mAP).

Method Used	Training Time	Inference Time	Mean Average Precision (mAP)
Yolov5s	1 iteration per second	0.249 s	99.1%
EfficientDet d0	2 iterations per second	0.03 s	94.3%

**Table 5:** Comparison of testing results when using various experimental speckle affected SMI signals having variable optical feedback conditions.

Method Used	Accuracy of Fringe Detection
Proposed (Yolov5)	99.1%
Proposed (EfficientDet)	94.3%
Bes et al. [48]	71.6%
Work under review [37]	66.18%

## Data Availability

The data can be obtained upon request.

## Abbreviations

The following abbreviations are used in this manuscript:

SMI	Self-Mixing Interferometry
OFI	Optical Feedback Interferometry
FD	Fringe Detection
VOF	Variable Optical Feedback
DNN	Deep Neural Network
GAN	Generative Adversarial Network
SNR	Signal to Noise Ratio
CNN	Convolutional Neural Network
GRNN	Generalized Regression Neural Network
LD	Laser Diode
PD	Photo Diode
AWGN	Additive White Gaussian Noise
BiFPN	Bi-directional Feature Pyramid Network
CSP	Cross Stage Partial
SSP	Spatial Pyramid Pooling

## References

[1] Taimre T., Nikolić M., Bertling K., Lim Y.L., Bosch T., Rakić A.D. (2015). Laser feedback interferometry: A tutorial on the self-mixing effect for coherent sensing. Adv. Opt. Photonics.

[2] Otsuka K. (2011). Self-mixing thin-slice solid-state laser metrology. Sensors.

[3] Li R., Hu Z., Li H., Zhao Y., Liu K., Tu Y., Du Z., Yu Q., Yu B., Lu L. (2022). All-fiber laser-self-mixing interferometer with adjustable injection intensity for remote sensing of 40 km. J. Light. Technol..

[4] Demir A.G., Colombo P., Norgia M., Previtali B. (2016). Evaluation of self-mixing interferometry performance in the measurement of ablation depth. IEEE Trans. Instrum. Meas..

[5] Zhao Y., Wang C., Zhao Y., Zhu D., Lu L. (2021). An all-fiber self-mixing range finder with tunable fiber ring cavity laser source. J. Light. Technol..

[6] Ali N., Zabit U., Bernal O.D. (2021). Nanometric vibration sensing using spectral processing of laser self-mixing feedback phase. IEEE Sens. J..

[7] Han D., Wang M., Zhou J. (2007). Self-mixing speckle in an erbium-doped fiber ring laser and its application to velocity sensing. IEEE Photonics Technol. Lett..

[8] Liu B., Ruan Y., Yu Y., Xi J., Guo Q., Tong J., Rajan G. (2018). Laser self-mixing fiber Bragg grating sensor for acoustic emission measurement. Sensors.

[9] Bertling K., Perchoux J., Taimre T., Malkin R., Robert D., Rakić A.D., Bosch T. (2014). Imaging of acoustic fields using optical feedback interferometry. Opt. Express.

[10] Di Cecilia L., Cattini S., Giovanardi F., Rovati L. (2016). Single-arm self-mixing superluminescent diode interferometer for flow measurements. J. Light. Technol..

[11] Perchoux J., Quotb A., Atashkhooei R., Azcona F.J., Ramírez-Miquet E.E., Bernal O., Jha A., Luna-Arriaga A., Yanez C., Caum J. (2016). Current developments on optical feedback interferometry as an all-optical sensor for biomedical applications. Sensors.

[12] Ottonelli S., Dabbicco M., De Lucia F., Di Vietro M., Scamarcio G. (2009). Laser-self-mixing interferometry for mechatronics applications. Sensors.

[13] Donati S., Martini G. (2019). 3D profilometry with a self-mixing interferometer: Analysis of the speckle error. IEEE Photonics Technol. Lett..

[14] Kliese R., Rakić A. (2012). Spectral broadening caused by dynamic speckle in self-mixing velocimetry sensors. Opt. Express.

[15] Merlo S., Donati S. (1997). Reconstruction of displacement waveforms with a single-channel laser-diode feedback interferometer. IEEE J. Quantum Electron..

[16] Bernal O.D., Zabit U., Jayat F., Bosch T. (2021). Toward an Estimation of the Optical Feedback Factor C on the Fly for Displacement Sensing. Sensors.

[17] Haider U., Zabit U., Bernal O.D. (2021). Variable Optical Feedback-Based Behavioral Model of a Self-Mixing Laser Sensor. IEEE Sens. J..

[18] Ozdemir S.K., Ohno I., Shinohara S. (2008). A comparative study for the assessment on blood flow measurement using self-mixing laser speckle interferometer. IEEE Trans. Instrum. Meas..

[19] Zabit U., Bernal O.D., Bosch T. (2012). Self-mixing laser sensor for large displacements: Signal recovery in the presence of speckle. IEEE Sens. J..

[20] Norgia M., Donati S., D’Alessandro D. (2001). Interferometric measurements of displacement on a diffusing target by a speckle tracking technique. IEEE J. Quantum Electron..

[21] Atashkhooei R., Royo S., Azcona F.J. (2013). Dealing with speckle effects in self-mixing interferometry measurements. IEEE Sens. J..

[22] Bernal O.D., Seat H.C., Zabit U., Surre F., Bosch T. (2016). Robust detection of non-regular interferometric fringes from a self-mixing displacement sensor using bi-wavelet transform. IEEE Sens. J..

[23] Arriaga A.L., Bony F., Bosch T. (2014). Speckle-insensitive fringe detection method based on Hilbert transform for self-mixing interferometry. Appl. Opt..

[24] Bernal O.D., Zabit U., Bosch T.M. (2014). Robust method of stabilization of optical feedback regime by using adaptive optics for a self-mixing micro-interferometer laser displacement sensor. IEEE J. Sel. Top. Quantum Electron..

[25] Siddiqui A.A., Zabit U., Bernal O.D., Raja G., Bosch T. (2017). All analog processing of speckle affected self-mixing interferometric signals. IEEE Sens. J..

[26] Mennel L., Symonowicz J., Wachter S., Polyushkin D.K., Molina-Mendoza A.J., Mueller T. (2020). Ultrafast machine vision with 2D material neural network image sensors. Nature.

[27] Sun J., Chang J., Wei Y., Zhang Z., Lin S., Wang F., Zhang Q. (2022). Dual gas sensor with innovative signal analysis based on neural network. Sens. Actuators Chem..

[28] Du Y.C., Stephanus A. (2018). Levenberg-Marquardt neural network algorithm for degree of arteriovenous fistula stenosis classification using a dual optical photoplethysmography sensor. Sensors.

[29] Tang Y., Chen M., Lin Y., Huang X., Huang K., He Y., Li L. (2020). Vision-based three-dimensional reconstruction and monitoring of large-scale steel tubular structures. Adv. Civ. Eng..

[30] Del Hougne M., Gigan S., Del Hougne P. (2021). Deeply subwavelength localization with reverberation-coded aperture. Phys. Rev. Lett..

[31] Zabit U., Shaheen K., Naveed M., Bernal O.D., Bosch T. (2018). Automatic detection of multi-modality in self-mixing interferometer. IEEE Sens. J..

[32] Wei L., Chicharo J., Yu Y., Xi J. Pre-processing of signals observed from laser diode self-mixing intereferometries using neural networks. Proceedings of the 2007 IEEE International Symposium on Intelligent Signal Processing.

[33] Ahmed I., Zabit U., Salman A. (2019). Self-mixing interferometric signal enhancement using generative adversarial network for laser metric sensing applications. IEEE Access.

[34] Barland S., Gustave F. (2021). Convolutional neural network for self-mixing interferometric displacement sensing. Opt. Express.

[35] Ge S., Kong X., Zhu D., Chen H., Lin Y., Wang X., Huang W. (2022). Robust Signal Extraction Based on Time-Frequency Joint Analysis and GRNN for a Laser SMI System. J. Light. Technol..

[36] Kou K., Wang C., Lian T., Weng J. (2020). Fringe slope discrimination in laser self-mixing interferometry using artificial neural network. Opt. Laser Technol..

[37] Khurshid S.S., Hussain W., Zabit U., Bernal O.D. (2022). Augmentation assisted robust fringe detection on unseen experimental signals applied to optical feedback interferometry using a deep network. TechRxiv.

[38] Plantier G., Bes C., Bosch T. (2005). Behavioral model of a self-mixing laser diode sensor. IEEE J. Quantum Electron..

[39] Tan M., Pang R., Le Q.V. Efficientdet: Scalable and efficient object detection. Proceedings of the IEEE/CVF Conference on Computer Vision and Pattern Recognition.

[40] GmbH S. A Pytorch Implementation of EfficientDet Object Detection. https://github.com/signatrix/efficientdet.

[41] Jocher G., Nishimura K., Mineeva T., Vilariño R. (2020). yolov5. *Code Repos*. https://github.com/ultralytics/yolov5.

[42] Redmon J., Farhadi A. YOLO9000: Better, faster, stronger. Proceedings of the IEEE Conference on Computer Vision and Pattern Recognition.

[43] Mekhalfi M.L., Nicolò C., Bazi Y., Rahhal M.M.A., Alsharif N.A., Maghayreh E.A. (2022). Contrasting YOLOv5, Transformer, and EfficientDet Detectors for Crop Circle Detection in Desert. IEEE Geosci. Remote. Sens. Lett..

[44] Bernal O.D., Zabit U., Bosch T. (2013). Study of laser feedback phase under self-mixing leading to improved phase unwrapping for vibration sensing. IEEE Sens. J..

[45] Fan Y., Yu Y., Xi J., Chicharo J.F. (2011). Improving the measurement performance for a self-mixing interferometry-based displacement sensing system. Appl. Opt..

[46] Osinski M., Buus J. (1987). Linewidth broadening factor in semiconductor lasers–An overview. IEEE J. Quantum Electron..

[47] Yáñez C., Royo S. (2020). Improvement of the signal-to-noise ratio in a low power self-mixing interferometer using a coupled interferometric effect. Opt. Express.

[48] Bes C., Plantier G., Bosch T. (2006). Displacement measurements using a self-mixing laser diode under moderate feedback. IEEE Trans. Instrum. Meas..

[49] Tang Y., Zhou H., Wang H., Zhang Y. (2023). Fruit detection and positioning technology for a Camellia oleifera C. Abel orchard based on improved YOLOv4-tiny model and binocular stereo vision. Expert Syst. Appl..

[50] Bernal O.D., Zabit U., Jayat F., Bosch T. (2020). Sub-*λ*/2 displacement sensor with nanometric precision based on optical feedback interferometry used as a non-uniform event-based sampling system. IEEE Sens. J..

